# 

**Published:** 1891-03

**Authors:** 


### SUBSCRIBE FOR THE

### International Dental Journal,

### The Organ of the Profession.

